# Semi-high-throughput detection of *Plasmodium falciparum* and *Plasmodium vivax* oocysts in mosquitoes using bead-beating followed by circumsporozoite ELISA and quantitative PCR

**DOI:** 10.1186/s12936-017-2011-9

**Published:** 2017-09-06

**Authors:** Wouter Graumans, Fitsum G. Tadesse, Chiara Andolina, Geert-Jan van Gemert, Karina Teelen, Kjerstin Lanke, Endalamaw Gadisa, Delenasaw Yewhalaw, Marga van de Vegte-Bolmer, Rianne Siebelink-Stoter, Isaïe Reuling, Robert Sauerwein, Teun Bousema

**Affiliations:** 1 Department of Medical Microbiology, Radboud University Nijmegen Medical Centre, Nijmegen, The Netherlands; 2 Armauer Hansen Research Institute (AHRI), Addis Ababa, Ethiopia; 3 Institute of Biotechnology, Addis Ababa University, Addis Ababa, Ethiopia; 4 Tropical and Infectious Diseases Research Center (TIDRC), Jimma University, Jimma, Ethiopia; 5 Department of Medical Laboratory Sciences and Pathology, Faculty of Health Sciences, Jimma University, Jimma, Ethiopia; 6 Department of Immunology and Infection, London School of Hygiene and Tropical Medicine, London, UK

**Keywords:** Transmission, Oocyst, Sporozoite, Anopheles, Infectivity, Gametocyte

## Abstract

**Background:**

The malaria infection status of mosquitoes is commonly determined by microscopic detection of oocysts on the dissected mosquito midgut. This method is labour-intensive, does not allow processing of large numbers of mosquitoes and can be challenging in terms of objective classification of oocysts. Here, a semi-high-throughput bead-beating ELISA method is proposed for detection of the circumsporozoite protein (CSP) followed by confirmation by quantitative PCR (qPCR).

**Methods:**

Cultured *Plasmodium falciparum* gametocytes were offered to *Anopheles stephensi* mosquitoes and examined by microscopy. After bead-beating, mosquito homogenate was examined by CSP-ELISA and 18S qPCR. As negative controls, mosquitoes that were offered a heat-inactivated gametocyte blood meal were used. The CSP-ELISA/qPCR methodology was applied to high and low-intensity infections of cultured *P. falciparum* gametocytes. A similar methodology optimized for *P. vivax* was used on mosquitoes that were offered blood from Ethiopian donors who were naturally infected with *P. vivax*.

**Results:**

There was considerable variation in CSP-ELISA signal and qPCR values in mosquitoes with low oocyst intensities. There was a strong agreement mosquito positivity by CSP-ELISA and by qPCR in mosquitoes that fed on cultured *P. falciparum* material (agreement 96.9%; kappa = 0.97) and naturally infected *P. vivax* parasite carriers [agreement 92.4% (kappa = 0.83)].

**Conclusions:**

The proposed bead-beating CSP-ELISA/qPCR methodology considerably increases throughput for the detection of mosquito infection. qPCR remains necessary to confirm infections in mosquitoes with low CSP-ELISA signal. This methodology may prove particularly useful for studies where very low mosquito infection prevalence is expected and study sites where experience with oocyst detection is limited.

**Electronic supplementary material:**

The online version of this article (doi:10.1186/s12936-017-2011-9) contains supplementary material, which is available to authorized users.

## Background

Although considerable progress has been made in malaria control during the last decades, malaria continues to pose a major public health burden, with ~212 million cases worldwide, particularly in sub-Saharan Africa [1]. Transmission reducing intervention strategies are considered of great importance for the enhanced control or elimination of malaria [2]. The transmission of malaria from human to mosquitoes depends on the presence of circulating mature sexual stages of the parasite, gametocytes, that are taken up by female *Anopheles* mosquitoes along with the blood meal that contains nutrients required for egg production. Once ingested, male and female gametocytes activate and fuse to form a zygote that develops into an ookinete. This motile form penetrates the mosquito midgut and differentiates into an oocyst. The oocyst matures over time and ruptures around day 11 post infection, to release hundreds to thousands of sporozoites, ending up in the salivary glands of the mosquito, ready to infect the subsequent new host [3, 4].

There are over 60 *Anopheline* mosquito species that are able to transmit malaria [5]. Traditionally, the infection status of mosquitoes is determined by microscopic detection of oocysts on the dissected mosquito midgut. This labour-intensive method requires skilled technicians and even then it can remain challenging to unequivocally classify oocysts or oocyst-like structures [6]. To increase throughput and improve objectivity of oocyst detection, several alternative approaches to microscopy have been proposed. In the standard membrane feeding assay (SMFA) that uses cultured gametocytes, throughput can be increased by using transgenic parasites expressing firefly luciferase during oocyst development [7]. In this quantitative luminescence based SMFA, individual or pooled mosquitoes are hand grinded and their relative light units (RLU’s) are measured by a micro plate reader. Compared to microscopic based readouts, a fivefold to tenfold increase in output is feasible with this approach [7].

For mosquito-feeding experiments that use naturally infected gametocyte carriers, alternative read-outs that can improve throughput compared to microscopy have also been proposed. Screening of individual mosquitoes to detect circumsporozoite protein (CSP) levels, the most abundant protein present in the oocyst/sporozoite stage from day 7 post-infection onwards, with the colourimetric enzyme-linked immunosorbent assay (ELISA) has recently been proposed as read-out for feeding assays [8–10]. Mosquitoes can be homogenized prior to CSP-ELISA by hand pestle grinding [3], resulting in a reliable, objective and efficient method for infection detection. In addition, an enhanced chemiluminescent slot blot immuno-assay (ECLSB) was recently presented as alternative detection method for CSP [11].

To increase throughput further, the current study tested an approach to increase the speed of time-limiting step of mosquito homogenization. For this methodology, mosquitoes in 96-deepwell plates were homogenized by adding silica beads and vigorous mechanical plate shaking, also known as bead-beating. A semi-high-throughput bead-beating CSP-ELISA method is presented for rapid screening of mosquitoes for the presence of oocysts/sporozoites. CSP-ELISA results were validated by qPCR targeting the 18S rRNA small subunit gene using the mosquito homogenate. This approach was optimized using mosquitoes fed on cultured *P. falciparum* gametocytes and subsequently applied to mosquitoes that were fed on naturally infected *P. falciparum* and *P. vivax* gametocyte carriers. The approach in the present work allows experimental data to be processed more rapidly and objectively, thus contributing to an increase in experimental output in studies that aim to increase understanding of malaria transmission.

## Methods

### Blood collection from parasite culture, naturally infected individuals and mosquito feeding

The Nijmegen *P. falciparum* 54 (NF54) strain [12], was cultured in an automated tipper system [13]. An infective blood meal was prepared as previously described [14]; after 14-days of parasite culture, 2% haematocrit and 0.3–0.5% gametocytes. Uninfected and infected but heat inactivated (2 h at 56 °C) blood was used as negative control, the latter allowing the assessment of DNA persistence in mosquitoes [15]. *A. stephensi* mosquitoes [16] (Sind-Kasur strain) were reared at 30 °C and 70–80% humidity, with a 12 h reverse day/night cycle. In vitro culture material was offered to 1–5 days old female mosquitoes using a glass membrane mini feeder system [14]. Unfed and partially fed mosquitoes were removed 30 min after feeding was completed. Fully fed mosquitoes were transferred to clean mini-cages and maintained with 5% sucrose solution at the rearing conditions. A fraction of mosquitoes (typically 20) were sacrificed between day 6 and 9 for oocyst detection after midgut staining (1% mercurochrome solution). Remaining mosquitoes were finally frozen on day 12 at −20 °C in tubes that contained silica gel beads that was covered with cotton [17] until processed for ELISA.

In the field, blood samples from individuals naturally infected with *P. falciparum* and *P. vivax* was used. This study, of which the main results will be published elsewhere, received ethics approval from the ethics review boards of Addis Ababa University (CNSDO/264/08/16), Jimma University (RPGC/395/06), Armauer Hansen Research Institute (PO52/14), The National Research Ethics Review Committee (310/109/2016) and the London School of Hygiene and Tropical Medicine (10628). Self-presenting patients at Adama Malaria Clinic were requested for participation in the study after the objectives, risks and benefits of the study were explained and written informed consent was sought. 5 mL venous blood was collected in Heparin and EDTA coated tubes (vacutainer) using Precision-Glide™ Multisample Needles (vacutainer) for mosquito feeding and molecular analyses, respectively.

*Anopheles arabiensis* colony mosquitoes were reared locally at 26–30 °C and 60–80% humidity at the insectary of the tropical infectious disease research center (TIDRC) of Jimma University at Sekoru. Blood was offered to 3–5 days old female mosquitoes using procedures identical to those described above. All mosquitoes were frozen on day 12 for ELISA.

### Bead beating of mosquitoes: standardizing beads for 96-deepwell plates and plate sealing

**Fig. 1:**
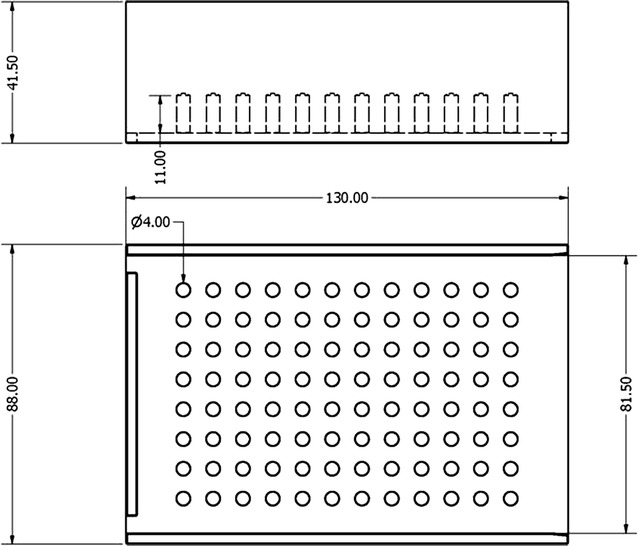
Custom-made 96 wells aluminum stand designed to harmonize the volume of beads in the plates. Each well had a volume of 142 mm^3^ beads

During the process of bead beating, silica beads were added to intact mosquitoes in PBS that were homogenized by vigorous mechanical plate shaking. Specifically, a 96-deepwell plate (CoStar 3958) was filled out with 1 mm zirconia 0.2 g 1 mm beads (Biospec) by using a custom-made aluminum stand with 96 holes of 142 mm^3^ each, to standardize the volume of beads between wells (Fig. 1). Tweezers were used to transfer individual frozen mosquitoes to wells and subsequently 100 µL of PBS was added. Three different sealing covers were tested for their bead beating suitability during mosquito homogenization: a disposable adhesive aluminum cover slip (Axygen, PCR-AS-200), a reusable silicone sealing mat (Porvair) and a plastic disposable adhesive sealing mat (Bio-Rad). After the adhesive sealing mat was applied, mosquitoes were beaten for 10 s (Biospec, Mini Beadbeater 96). A shorter duration of beating (5 s) was considered disadvantageous based on a modest set of preparatory experiments (Additional file 1: Figure S1).

Plates were subsequently centrifuged at 2000 rpm for 1 min to spin down the homogenate and beads. The sealing mat was removed and 150 µL of buffer (1.66% sarkosyl in PBS-0.083% Tween) was added using a multichannel pipet, to acquire a total sample volume of 250 µL. Plates were directly used in ELISA or stored with a new sealing mat at −20 °C until being processed. In preparatory experiments, several buffers were tested. An overview of findings is given in the supporting information (Additional file 1: Table S1).

### Circumsporozoite protein enzyme linked immune-sorbent assay (CSP-ELISA)

#### *Plasmodium falciparum*

ELISA plates (thermo scientific) were coated with 100 μL of 5 μg/mL 3SP2 (Nijmegen, Netherlands) in PBS and incubated for 3 h at room temperature (RT) [9]. Plates were washed 3 times with PBS; subsequently 150 μL blocking buffer (5% dried skimmed milk in PBS) was added. After 1 h incubation at RT plates were washed three times with PBS and 50 µL of mosquito homogenate was transferred to the ELISA plate. Multiple blank wells (no homogenate) and pooled negative control wells (homogenate from uninfected mosquitoes) were used on each plate together with an eight step (threefold dilution) standard curve of recombinant CSP (Gennova, 0.02 μg/mL). Plates were incubated overnight at 4 °C and washed four times with PBS the following day. A 100 µL conjugate monoclonal antibody (3SP2 HRP, 0.5 μg/mL in PBS—0.05% tween—1% milk) was added to the wells and left for 3 h at RT. Plates were subsequently washed four times with PBS. A 100 µL of substrate (TMB, SURMODICS) was added to the wells, and left for 20 min at RT. Finally, 50 µL of 0.2 M H_2_SO_4_ was added to stop the reaction. Absorbance was read at 450 nm to determine optical density values using iMark™ microplate absorbance reader (Bio-Rad).

#### *Plasmodium vivax* and mixed species infections with *P. falciparum*

The *P. vivax* CSP protein contains a variable central region that is composed of two nanopeptides that repeat in tandem and result in the two major molecular variants of the protein, VK210 and VK247 [18]. Local and global variations have been reported in the distribution of the two variants [19, 20] and also variation in vector susceptibility towards the two forms [21]. One of the challenges in investigating the anti-CSP protein immunity in *P. vivax* samples is the consumption of twice the volume required for a *P. falciparum* assay, which may exhaust the homogenate before subsequent molecular tests can be performed. This is particularly pressing when sample needs to be analysed for mixed species infections (and thus three ELISAs that each require 50 µL homogenate need to be run). To economize sample usage an approach was tested where a single 50 µL volume was transferred from one ELISA reaction plate to the next (Additional file 1: Figure S2). In this approach, the *P. falciparum* plate was coated with 5 µg/mL of the capture monoclonal antibody overnight at 4 °C. Plates were washed three times with PBS, followed by blocking. 50 µL of mosquito homogenate, obtained as described above, was incubated on the *P. falciparum* plate for 2 h at RT. At the same time, the VK210 plate was coated with its monoclonal antibody for 30 min at RT and washed three times with PBS, followed by blocking. At the end of the 2 h the homogenate was transferred with multichannel pipettes to the VK210 plate and incubated at RT for 2 h. The *P. falciparum* plate was washed three times and incubated with HRP-tagged monoclonal for 1 h at RT. At the same time, the VK247 plate was coated with the capture antibody for 30 min at RT that was followed by three times washing and subsequent blocking. Mosquito homogenate was transferred to VK247 plates and incubated for 2 h at RT. The VK210 plates were washed three times and incubated with HRP-tagged monoclonal antibody for 1 h at RT. The *P. falciparum* plate was washed four times and 100 µL of TMB was incubated for 20 min followed by addition of 50 µL stop solution. The VK247 plates were washed three times and incubated with HRP-tagged monoclonal antibody for 1 h at RT. The VK210 and VK247 plates were subsequently washed four times with PBS and incubated with TMB at RT for 20 min and reaction was stopped using 50 µL of 0.2 M H_2_SO_4_. In all cases 150 μL of blocking buffer (5% skimmed milk) was used to block plates for 1 h at RT.

All ELISA plates included mosquitoes fed on negative blood and threefold serial dilutions of positive controls of the respective reaction. To test for cross-reactivity, each plate also contained positive controls of the other two targets. Absorbance was read for all plates at 450 nm to determine optical density values using iMark™ microplate absorbance reader (Bio-Rad).

### DNA extraction and confirmation by 18S qPCR

After ELISA was performed the remaining mosquito homogenate from each sample was stored at −20 °C until the results from the ELISA were available. Mosquito homogenate was incubated overnight at 65 °C with Proteinase K (QIAGEN). DNA was extracted based on magnetic bead technology, with the automated MagNaPure LC instrument (Roche) using MagNaPure LC DNA Isolation kit (Roche, Basel, Switzerland). The standard extraction protocol was followed to elute DNA in 100 μL. DNA eluate was either immediately run on qPCR or stored at 4 °C for a few hours or at −20 °C for longer-term storage.

The presence of *P. falciparum* and *P. vivax* parasites was tested by 18S rRNA small subunit gene based qPCR, using primer and probe sequences described in Hermsen et al. [22] and Wampfler et al. [23], respectively, with minor modifications. Briefly, for the *P. falciparum* qPCR 5 μL of extracted DNA was used as template in a total reaction volume of 20 μL that consisted of 100 nM probe and primer concentration [24]. For *P. vivax* qPCR 2μL of DNA input was run in 12 μL final reaction volume that consisted of 110 nM probe and 833 nM primer concentration. Probes were from life technologies (applied biosystems) and primers were from SIGMA ALDRICH. All reactions were run using hard-shell 96 well PCR plate (Bio-Rad) and TaqMan^®^ fast advanced master mix (2X) (applied biosystems) and analyzed on the CFX96™ real-time PCR detection system (Bio-Rad).

### Assay sensitivity: detection of single *P*. *falciparum* oocyst

To investigate the ability of ELISA in detecting parasites in mosquitoes infected with single oocysts, a direct comparison between microscopy and ELISA was performed. Cultures of NF54 diluted gametocytes were fed to *A. stephensi* mosquitoes. Seven days post infection 20 mosquitoes were dissected from each batch to assess presence of oocysts following standard mercurochrome staining. Only batches of mosquitoes with low oocysts counts (45–50% prevalence; 1–4 oocysts) were selected. From these batches, additional mosquitoes were collected on day 10 and dissected in PBS. Dissection on day 12 was considered undesirable since oocysts may have ruptured or may be too fragile to dissect without initiating rupture. Midguts with 1–5 oocysts were carefully moved from the slide with a fine needle into a 100 µL PBS containing tube. Head, thorax and abdomen from each mosquito were also added to each tube to obtain the same material as used in the ELISA of undissected mosquitoes. Needles and tweezers were sterilized each time between dissections with 70% ethanol. Negative midguts and uninfected blood fed mosquitoes were kept. The combined material from individual mosquitoes was homogenized by bead beating in tubes, following the same beating protocol.

### Analysis

Positivity in the CSP-ELISA was defined as the mean optical density of a group of negative blood fed mosquitoes plus three standard deviations. CSP-ELISA positivity was analysed as dichotomous variable; optical density was used at a continuous scale, including both positive and negative samples. The threshold for positivity in the *P. falciparum* and *P. vivax* qPCR was set at a CT value of 35. qPCR positivity was analysed as dichotomous variable and as continuous variable (CT value), including positives only. The following questions were addressed: (i) what is the best sealing cover for bead-beating. This was assessed by visually inspecting whether the seal was intact and investigating potential leakage by staining the volume added to the plates blue. (ii) What is the appropriate negative control for the CSP-ELISA. For this blood-fed and unfed mosquitoes were compared on days 2 and 12 post feeding using the mean OD of individually processed mosquitoes by Student’s t test and the proportion of ELISA positive mosquitoes by Fisher’s Exact test. (iii) Can CSP-ELISA detect infections with similar sensitivity compared to qPCR. This was assessed by processing dissected and undissected mosquitoes, using mosquitoes that fed on culture material and blood from naturally infected individuals. (iv) For *P. vivax* and *P. falciparum* mixed infections: does sequential use of the same mosquito homogenate from one ELISA to the subsequent one affect CSP-ELISA results? For this, a comparison was made between the OD value of mosquito homogenate that was used in single CSP-ELISA assays with the OD value obtained after using the same homogenate sequentially in several ELISAs. Comparisons were done by non-parametric paired tests.

## Results

### Plate sealing

**Fig. 2:**
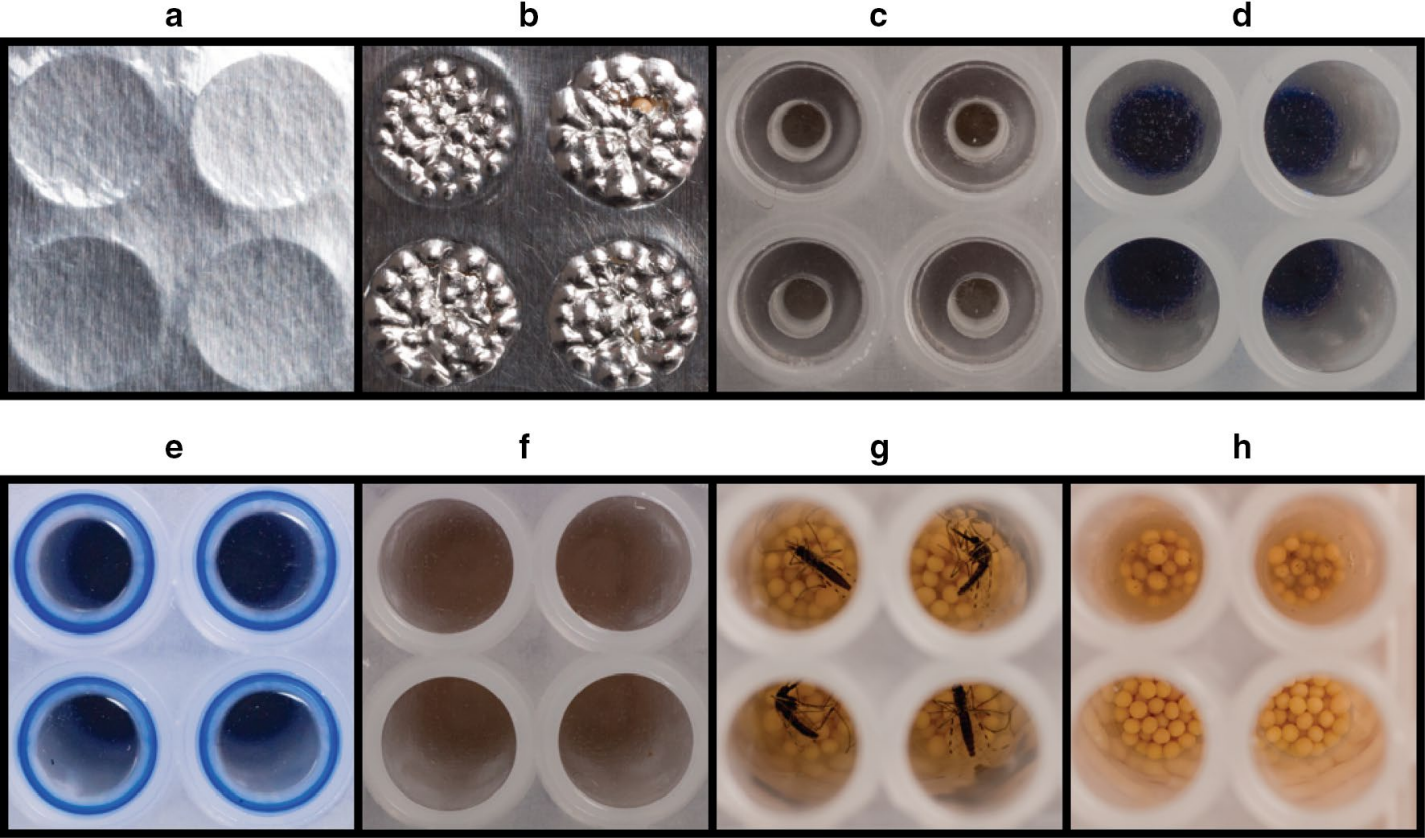
Plate sealing covers. The disposable adhesive aluminium cover slip before bead beating (**a**) and after bead beating (**b**). The reusable silicon sealing mat (**c**). The plastic disposable adhesive sealing mat (**d**), after bead beating (**e**) and spun down by centrifugation (**f**). Beads were prepared with beads and PBS (**h**, *lower two wells*), mosquitoes were added (**g**), and homogenized by bead beating for 10 s (**h**, *upper wells*)

Three different plate sealing covers were evaluated (Fig. 2). The disposable adhesive aluminium cover slip was not resistant to the force of the beats before (Fig. 2a) and after bead beating (Fig. 2b). The reusable silicon sealing mat (Fig. 2c) (Porvair) was resistant to bead beating, however DNA contamination (detected by 18S qPCR) was observed despite thorough cleaning with bleach between usages. The plastic disposable adhesive sealing mat (Bio-Rad) (Fig. 2d) proved to be the best cover for the current purposes. After bead-beating, no effect of the beads on the sealing was observed. Potential leakage was evaluated by staining PBS blue with Giemsa (Fig. 2d). After beating (Fig. 2e) no leakage was seen, plates were spun down (Fig. 2f) before removing the sealing cover. After filling out the plates with beads and addition of PBS (Fig. 2h, lower two wells), mosquitoes were added to the wells (Fig. 2g), and homogenized for 10 s by bead beating (Fig. 2h, upper wells).

### The appropriate negative control for *Plasmodium falciparum* CSP-ELISA and qPCR

**Fig. 3:**
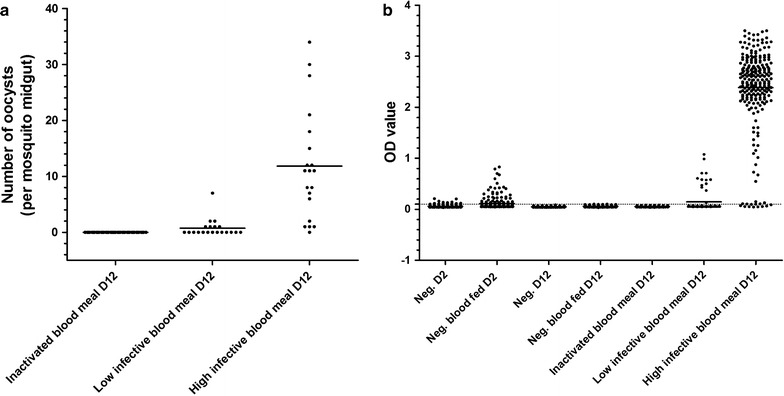
Oocyst dissection data compared to *P. falciparum* CSP-ELISA results. The number of oocysts per mosquito read by microscopy on day-12 (d12) post feeding for the inactivated blood meal and the low and high infected blood meal (**a**). ELISA OD measurements for: negative mosquitoes collected on day-2 (d2) and d12 (with and without a blood meal on day-0 (d0), and the inactivated *Plasmodium falciparum* blood meal, a low infection and a high infection collected on d12 (**b**). *Points* representing single mosquitoes, *dashed lines* show the cut-off value for positivity and *bars* the mean OD value

For *P. falciparum,* all infected blood and gametocyte-positive but heat-inactivated blood fed mosquitoes were dissected and microscopically examined for oocysts. Infection prevalence in the infected mosquitoes for the high infection was 95% (n = 20, 0–34 oocysts, SD 9.9) and 35% for the low infection (n = 20, 0–7 oocysts, SD 1.6). The heat inactivated group showed no oocysts development (n = 60). For each experimental condition a proportion of the undissected *A. stephensi* mosquitoes were collected on day 12 post feeding and analysed by ELISA (Fig. 3; Table 1). The OD average of 240 negative blood-fed mosquitoes on day 12 plus three times the standard deviation was determined as cut-off value for ELISA positivity (OD = 0.103).

**Table 1:** Dissection and CSP-ELISA results for mosquitoes without a blood meal, with a non-infected blood meal, with an inactivated gametocyte blood meal or an infectious gametocyte blood meal

	Day	Mean oocysts + range	Microscopy infection prevalence, % (n/N)		Mean optical density + standard deviation	CSP-ELISA infection prevalence, % (n/N)	
Negative	D2	N/A	–	–	0.0583 (0.0230)	1.3 (3/240)	
Negative (blood meal)	D2	N/A	–	–	0.1113 (0.1201)	14.2 (34/240)	
Negative	D12	N/A	–	–	0.0507 (0.0080)	0.0 (0/240)	
Negative (blood meal)	D12	N/A	–	–	0.0601 (0.0143)	0.0 (0/240)	
Inactivated gametocyte blood meal	D12	0 (0)	0.0	(0/60)	0.0548 (0.0077)	0.0 (0/240)	
Low infectious gametocyte blood meal	D12	0.8 (0–7)	35.0*	(7/20)	0.1437 (0.2217)	16.3** (13/80)	
High infectious gametocyte blood meal	D12	11.9 (0–34)	95.0	(19/20)	2.386 (0.7820)	93.8 (225/240)	

*Day* day of mosquito collection after feeding

*Confidence interval microscopy for low infectious meal: 35.0 (95% CI 15.3–59.2)

**Confidence interval ELISA for low infectious meal: 16.3 (8.9–26.2)

The OD and positivity in the CS-ELISA was compared with the CT value and positivity in the qPCR (Additional file 1: Table S2). To assess DNA persistence, qPCR was performed on a batch of mosquitoes that were fed a negative blood meal with heat inactivated gametocytes (n = 32), as positive control on a low infected batch of mosquitoes (n = 32). The CT cut-off value for positivity was set at 35. One OD negative heat-inactivated sample was positive in qPCR (OD = 0.065; CT = 28.58), and for the low infected mosquito batch, one ELISA-positive mosquito was qPCR negative (OD = 0.132; CT = 36.46).

### *Plasmodium falciparum* and *P. vivax* CSP-ELISA assay sensitivity

**Fig. 4:**
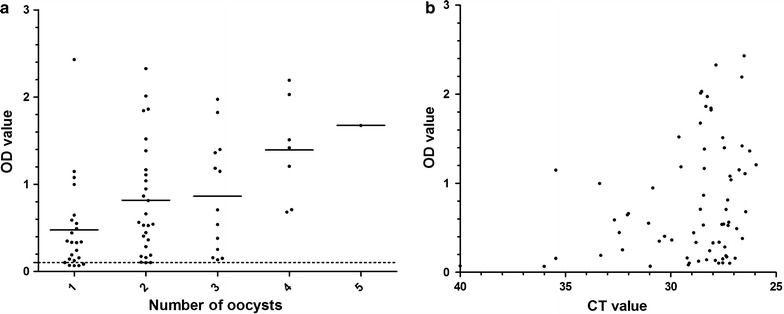
Assay sensitivity detection of *P. falciparum* oocysts. OD values of CSP-ELISA on 71 *P. falciparum* infected mosquitoes with oocyst numbers between 1 and 5 (**a**). Presented on the *X-axis* is qPCR CT values versus CSP-ELISA OD measurements in the *Y-axis* (**b**). *Bars* are representing the mean

Dissected and microscopy examined mosquitoes were used to directly investigate the ability of CSP-ELISA to detect parasites in mosquitoes with low oocyst numbers. For this purpose, midguts with 1–5 oocysts (n = 71) were selected on day 10 post feeding (prior to possible oocyst rupture) and processed for CSP-ELISA (Fig. 4). Almost all the mosquitoes with low oocyst densities were CSP-ELISA positive (91.5%; 65/71), but there was considerable variation in OD values. The average OD for a mosquito with one oocyst was 0.478 (n = 23, range 0.067–2.431, SD 0.5349), for two oocysts was 0.837 (n = 27, range 0.102–2.327, SD 0.6457); for three oocysts 0.862 (n = 13, range 0.134–1.974, SD 0.6549); for four oocysts 1.392 (n = 7, range 0.681–2.193, SD 0.5873). The OD value for a single mosquito with five oocysts was 1.675. There was a statistically significant positive association between CSP-ELISA OD value and the number of oocysts across this range (β = 0.23, se(β) 0.11; p = 0.04) (Fig. 4a). For mosquitoes processed by qPCR, there was a negative correlation between CSP-ELISA results (higher indicating higher infection burden) and qPCR CT values (lower indicating higher infection burden; Spearman correlation coefficient −0.25, p = 0.035) (Fig. 4b).

The OD and positivity in the CSP-ELISA was compared with the CT value and positivity in the qPCR (Additional file 1: Table S1). The qPCR CT cut-off value for positivity was set at 35. One CSP-ELISA negative heat-inactivated sample was positive in qPCR (OD 0.065; CT = 28.58), and for the low infected mosquito batch, one ELISA-positive mosquito was qPCR negative (OD 0.132; CT = 36.46). When combining 96 mosquitoes included in in vitro culture experiments with infectious material (n = 64) or heat-inactivated material (n = 32) that were tested in both CSP-ELISA and qPCR, the level of observed agreement according to Cohen’s Kappa was 96.88% (kappa = 0.973).

**Fig. 5:**
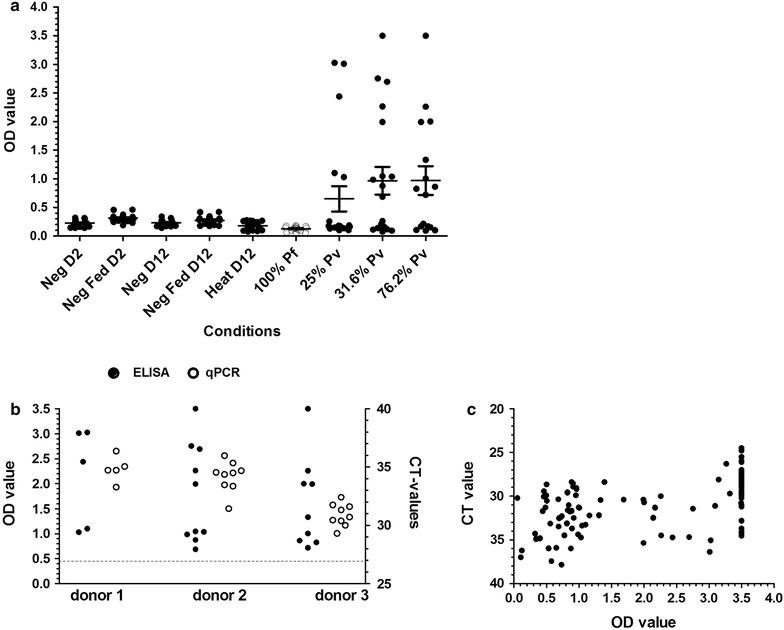
Performance of negative controls in *P. vivax* CSP ELISA and agreement between CSP-ELISA and qPCR. Optical density values are plotted for *A. stephensi* mosquitoes that received or did not receive a blood meal prior to processing on D2 (day 2) or D12 (day12), high gametocyte culture *P. falciparum* infected blood fed mosquitoes (100%) and mosquitoes that fed on three microscopy positive *P. vivax* carriers that were processed on D12 (**a**). The relative signals from the CSP-ELISA (*filled circles*) and qPCR data (*clear circles*) are presented for three donors (**b**). CSP-ELISA and corresponding qPCR data are shown for 198 mosquitoes that fed on 55 naturally infected *P. vivax* parasite carriers (**c**). *Dotted line* (**b**) indicates the OD cut-off value. % for the three donors in (**a**) indicate the percentage of infected mosquitoes

Background *P. vivax* ELISA OD values for different *A. stephensi* negative controls were low in comparison with OD values for *A. arabiensis* mosquitoes that were fed blood meals from naturally infected *P. vivax* gametocyte carriers (Fig. 5a). Both CSP-ELISA OD values and qPCR CT values showed clear positivity but considerable variation for three donors with *P. vivax* gametocyte densities of (gametocytes/µL) of 2654, 497, and 13,616 (Fig. 5b). In total 25 mosquitoes were processed by both methods (Fig. 5b). When CSP-ELISA data and qPCR were compared for 198 mosquitoes that were examined as part of a larger study (Tadesse, *in preparation*), a strong negative correlation between signal intensity in both assays (Spearman correlation coefficient −0.51, p < 0.001) (Fig. 5c) was observed and the level of observed agreement between CSP-ELISA positivity and qPCR positivity according to Cohen’s Kappa was 92.42% (kappa = 0.826).

### Sequential use of the same mosquito homogenate in *P. falciparum* and *P. vivax* CSP-ELISA

**Fig. 6:**
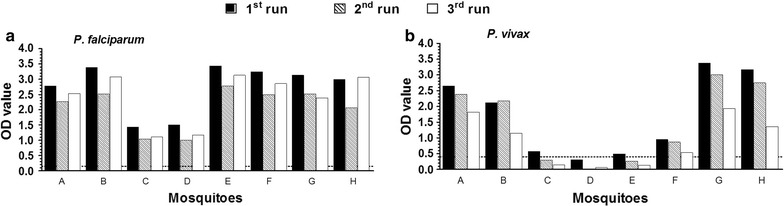
Sequential usage of the same volume for subsequent ELISA targets. *P. falciparum* (**a**) and *P. vivax* (VK210) (**b**) ELISA assay results where the same 50 µL homogenate was transferred from the 1st run assay (*dark bars*) to the 2nd (*hatched bars*) and subsequently to the 3rd (*clear bars*) is presented. Indicated in the *Y-axes* are OD values from absorbance reading at 450 nm and *X-axes* are individual mosquitoes. *Dotted line* (**b**) indicates the OD cut-off value

The mosquito bead-beating was optimized at 100 µL PBS and a later addition of 150 µL grinding buffer, which gives an approximate final volume of 200 µL for further analyses as a fraction of the total volume adheres to the beads. A single 50 µL homogenate was used in both *P. falciparum* and *P. vivax* experiments by transferring the homogenate from one reaction to the next. Signal generated from the *P. falciparum* assays (Fig. 6a) was generally stable when it was run first, second or third in order. The *P. vivax* (VK210) signal was stable between the first and second runs but lower OD values were observed when this actual target was the third ELISA performed. This relative loss in signal intensity resulted in some CSP-ELISA positive mosquitoes with OD values just above the threshold for positivity to be considered negative when the homogenate was tested as 2nd or 3rd ELISA (Fig. 6b). None of the tested samples were found positive with VK247 variant and it was difficult to investigate the effect of the order of the experiment on this target. Furthermore, cross-reactivity tests indicated that there was no inter-species cross-reactivity (between the *P. falciparum* and *P. vivax* targets) but the there was a strong cross-reactivity between the positive controls of VK210 and VK247.

## Discussion

In the present study, a mosquito bead-beating methodology on frozen specimens is presented to determine *P. falciparum* and *P. vivax* oocyst infections in mosquitoes by CSP-ELISA followed by qPCR confirmation. This methodology increases assay throughput and provides objective mosquito infection estimates for both in vitro experiments and mosquito feeding assays on naturally infected individuals. This study expanded upon a previous study that proved CSP can reliably be detected in mosquitoes 8 days post infection by ELISA and enhanced chemiluminescent slot blot immuno-assay (ECLSB) [3, 11]. Following bead-beating of mosquitoes that fed on either *P. falciparum* gametocyte culture material or blood from *P. vivax* gametocyte carriers, CSP-ELISA and 18S based qPCR identify the same mosquitoes as oocyst positive.

There is considerable interest in understanding the human infectious reservoir for malaria, defined as the proportion of the population capable of infecting mosquitoes. A large proportion of malaria-infected individuals harbours low-density parasites [25] with accompanying low gametocyte densities [26] that are associated with low but non-negligible mosquito infection rates [27]. Low mosquito infection rates necessitate a large number of mosquito observations for precision. The complexity of mosquito read-outs is increased by the co-endemicity of *P. falciparum* and *P. vivax* in many malaria endemic settings [28]. In regions where both species occur, mosquitoes may become infected with both *P. falciparum* and *P. vivax* [29] although it is nearly impossible to convincingly identify the species origin of low-oocyst infections. A direct approach to differentiate between the two species is molecular investigation. The ELISA methodology presented in this work has considerable advantages over direct molecular testing in terms of throughput and cost. Mixed infections were detected by ELISA using the same volume of 50 µL by sequentially transferring the homogenate from one reaction to the next. During these sequential reactions there was a small loss in signal that may affect the detectability of low density *P. vivax* infection, but it generally seemed possible to test the same mosquito homogenate in three ELISAs sequentially.

The current study used three approaches to confirm the presence of infections in CSP-ELISA positive mosquitoes. Firstly, the infection prevalence was assessed by microscopy in mosquitoes from the same cage as those processed by CSP-ELISA and qPCR. Especially in low-density infections, there is considerable random variation in infection rates between groups of mosquitoes, which is also evident in the current study where at low infection prevalence CSP-ELISA and microscopy estimated different infection rates, with overlapping confidence intervals [30]. Uncertainties in these indirect comparisons were circumvented by two additional direct comparisons. CSP-ELISA was directly performed on dissected and microscopically examined mosquito guts. This required oocyst detection without mercurochrome, since this affects CSP-ELISA signal (Stone et al., unpublished findings during experiments for [3]). Oocyst detection without staining is cumbersome and even with expert microscopy small oocysts may be missed. Moreover, removing infected guts from under cover slips for subsequent homogenization and processing can result unintentional oocyst rupture. Observed was that the majority of low oocyst infections were nevertheless CSP-ELISA and qPCR positive. The association between CSP-ELISA signal intensity and oocyst density was weak, which may reflect variable growth rates of oocysts [7, 31] that may result in a highly variable number of *P. falciparum* sporozoites developing from a single. Importantly, CSP-levels [30] and DNA copies [15] increase over time during sporogonic development. Since sporozoite density (rather than simple presence) may be an important determinant in determining the likelihood of achieving secondary infections in humans [32], it is worth exploring whether CSP-ELISA or qPCR signal can reliably quantify the productivity of oocysts in future studies.

Lastly, qPCR was used to confirm CSP-ELISA results using the same mosquito homogenate. With this approach excellent agreement was observed between CSP-ELISA infection prevalence and qPCR parasite prevalence. Using cultured gametocytes, an agreement rate of 96.88% (kappa = 0.973) was observed for *P. falciparum.* In natural *P. vivax* infections, a similar high agreement rate was observed of 92.42% (kappa = 0.826).

## Conclusion

A semi-high-throughput methodology was presented for the assessment of *P. falciparum* and/or *P. vivax* infected mosquitoes based on mosquito homogenization by bead-beating followed by CSP-ELISA and confirmed by qPCR. Results indicate that this method can be used to replace oocyst reading by microscopy with similar infection prevalence estimates, for single or mixed species *Plasmodium* infections, with less subjectivity and with more flexibility in processing (frozen) mosquito material. An estimate of the approximate time required for processing and screening mosquitoes for infection prevalence by the current CSP-ELISA/qPCR based method in comparison with traditional dissection-microscopy is given in the supporting information (Additional file 1: Table S3) The current approach also allows homogenate usage for other molecular assessments, for example of clonal complexity [33] and parasite resistance markers [34] and may prove particularly useful for study sites where experience with oocyst detection is limited or very low mosquito infection prevalence is expected. In such settings, mosquitoes may be collected after mosquito feeding experiments and processed in a well-equipped laboratory for an objective and scalable assessment of mosquito infection rates.

## Additional file

**Additional file 1: Table S1.** Observations with different buffers for mosquito homogenisation. **Table S2.** Experimental infection data CT values by qPCR vs Optical Density in CSP-ELISA. **Table S3.** Overview of processing time of the four methods compared in seconds per 80 mosquitoes. **Figure S1.** Protocol for mixed infection detection using the same 50 µl volume. Homogenate is transferred from the prior experiments to the subsequent ones. It is important that every step is timed and thought ahead on when to start the different steps. **Figure S2.** Processing of mosquitoes by CS-ELISA when tested for both *P. falciparum* and *P. vivax.*

## Abbreviations

ELISA: colometric enzyme-linked immunosorbent assay
CSP: circumsporozoite protein
CT: cycle threshold
DNA: deoxyribonucleic acid
ECLSB: enhanced chemiluminescent slot blot immuno-assay
qPCR: quantitative polymerase chain reaction
OD: optical density
RLU’s: relative light units
rRNA: ribosomal ribonucleic acid
RT: room temperature
SMFA: standard membrane feeding assay
