# Supplementary material for: Semi-high-throughput detection of Plasmodium falciparum and Plasmodium vivax oocysts in mosquitoes using bead-beating followed by circumsporozoite ELISA and quantitative PCR
Source: Malar J. 2017 Sep 6;16:356. doi: 10.1186/s12936-017-2011-9 (PMC5588624; doi:10.1186/s12936-017-2011-9)
Supplement: Supplementary file 1 — Additional file 1: Table S1. Observations with different buffers for mosquito homogenisation. Table S2. Experimental infection data CT values by qPCR vs Optical Density in CSP-ELISA. Table S3. Overview of processing time of the four methods compared in seconds per 80 mosquitoes. Figure S1. Protocol for mixed infection detection using the same 50 µl volume. Homogenate is transferred from the prior experiments to the subsequent ones. It is important that every step is timed and thought ahead on when to start the different steps. Figure S2. Processing of mosquitoes by CS-ELISA when tested for both P. falciparum and P. vivax. [file 12936_2017_2011_MOESM1_ESM.pdf]

Supporting Table S1. Observations with different buffers for mosquito homogenisation

Buffer for bead beating	Observation	Considered appropriate
PBS + sarcosyl-10	Foam development during beating	No, sarcosyl is better added after homogenisation
PBS + protease inhibitor cocktail kit (Thermo Scientific, #78410)	Appropriate CSP-ELISA results, appropriate DNA extraction results	No, rapid processing of mosquitoes may obviate the need for protease inhibitor
PBS without protease inhibitor cocktail kit (Thermo Scientific, #78410)	Appropriate CSP-ELISA results, appropriate DNA extraction results	Yes, sarcosyl should be added to homogenate prior to ELISA, rapid processing of mosquitoes obviate the need for protease inhibitor

Supporting Table S2. Experimental infection data CT values by qPCR vs Optical Density in CSP-ELISA. Mosquitoes infection prevalence for a high and low infectious blood meal was determined by microscopy and compared to OD and positivity in the CS-ELISA and CT-values and positivity in the qPCR. Positive samples in ELISA, defined as the mean optical density of a group of negative blood fed mosquitoes plus three standard deviations and qPCR, qPCR was set at a CT value of 35, are shown in green. Negative samples in red. Samples highlighted in yellow are conflicting, either ELISA or qPCR is positive, the other negative.

Heat inactivated<br>Microscopy: 0% infection (n=20)		Low infection<br>Microscopy: 35% infection (n=20)		High infection<br>Microscopy: 100% infection (n=20)	
CSP-ELISA OD<br>0% infection (n=32)	CT qPCR<br>3% infection (n=32)	CSP-ELISA OD<br>40.5% infection<br>(n=32)	CT qPCR<br>37.5% infection<br>(n=32)	CSP-ELISA OD<br>96.9% infection<br>(n=32)	CT qPCR<br>100% infection<br>(n=32)
0,065	28,58	0,056	-	3,316	29,61
0,051	-	0,47	29,20	3,284	27,15
0,077	-	0,065	-	2,735	27,36
0,047	-	0,708	28,30	3,200	27,13
0,074	-	0,429	28,64	2,729	28,30
0,084	-	0,058	-	2,424	27,14
0,059	43,21	0,056	-	2,425	28,89
0,065	-	0,055	36,39	2,559	26,17
0,051	-	0,132	36,46	1,603	27,90
0,082	-	0,574	28,21	2,268	26,78
0,076	-	0,071	37,65	2,186	28,04
0,063	-	0,055	-	2,496	27,79
0,054	-	0,048	-	2,866	26,08
0,054	37,50	0,709	28,23	2,315	27,58
0,06	-	0,368	30,15	2,148	28,55
0,052	38,92	0,06	36,53	2,446	28,17
0,046	-	1,077	26,26	3,058	27,04
0,068	35,75	0,068	36,46	2,34	28,20
0,054	-	0,054	36,41	2,326	29,49
0,063	-	0,065	-	2,42	29,16
0,045	36,38	0,582	29,93	2,98	28,29
0,062	-	0,065	-	2,039	28,12
0,063	-	0,061	34,89	2,11	28,59
0,051	-	0,607	27,95	2,788	27,57
0,042	-	0,059	33,26	2,981	27,46
0,061	36,77	0,062	34,42	0,091	28,32
0,059	-	0,986	26,88	3,003	28,43
0,046	37,77	0,049	-	2,789	28,71
0,051	43,20	0,057	36,32	2,361	27,45
0,056	-	0,059	36,24	3,02	27,83
0,052	36,71	0,501	28,50	2,535	27,57
0,049	-	0,583	28,91	2,978	28,47

Supporting Table S3. Overview of processing time of the four methods compared in seconds per 80 mosquitoes.

Method (per 80 mosquitoes)	Time (s)	Total time (s)
Mosquito dissection + microscopy reading	7400	7400
Preparation of tubes and addition of mosquitoes + Pestle grinding (motor driven) + ELISA (single channel pipette)	600 + 800 + 2600	4000
Preparation of plate and addition of mosquitoes + Bead beating + ELISA (multichannel pipette)	480 + 100 + 2400	2980

For each method a total of 80 mosquitoes were processed. Time starts after collecting 80 mosquitoes from each cage.

Microscopy

- Preparation of a slide with mercurochrome (2 mosquitoes per slide), removal of an individual mosquito, dissection and removal of midgut in mercurochrome, discarding carcass.

= Approx. 1 minute per mosquito.

- Storage for 10 minutes in mercurochrome (performed overlapping – so that the five-ten minutes it takes to stain a dissected gut can be filled with further dissections)

= No additional time

- Reading oocysts

= Approx. 30 seconds (appropriate for low intensity infections, >1 minute for high intensity infections).

- Additional time to copy up results

= 15 minutes

80 mosquitoes in approx. 124 minutes

Pestle grinding (motor) or bead-beating

Label tubes and add mosquitoes to eppendorf, or label plate and add mosquitoes to wells

= 4 minutes or 1.5 minutes

Pestle grinding (motor driven) = 7 seconds per mosquito

Bead beating = 10 seconds per plate (80 mosquitoes)

CSP-ELISA

Preparing coating and buffers

= 10 minutes

Coating ELISA plate, adding sample (3 hours incubation)

= 5 minutes (incubation = no additional time)

Washing plate 3 times + addition of blocking buffer

= 10 minutes

Incubation 1 hour

= no additional time

Preparing standard curve of recombinant CSP

= 5 minutes

Plate incubation overnight at 4°C

= no additional time

Adding monoclonal and three hours incubation

= 1 minutes (incubation = no additional time)

Washing plate 4 times

= 10 minutes

Adding substrate

= 1 minutes

Stop reaction

= 1 minutes

Read absorbance

= 2 minutes

80 mosquitoes in approx. 45 minutes

When bead beating was performed in a single 96-deepwell plate and a multichannel pipette was used to transfer material, an ~ 2.5 fold increase in throughput, compared to traditional microscopic evaluation can be achieved.

Supporting Figure S1. The effect of homogenization method on estimated infection prevalence.

In four independent experiments 69, 81, 87 and 81 were available to test homogenization methods. Thirty mosquitoes were processed for infection detection by microscopy on day 7 post infection (open bars). Remaining mosquitoes were divided in 3 batches with equal mosquito numbers (i.e. 13, 17, 19, 13 per batch for experiments 1, 2, 3 and 4, respectively) and processed by pestle grinding (hatched bars) and bead beating for 5 seconds (grey bars) or 10 seconds (black bars). Bead beating for 10 seconds consistently gave similar or higher infection prevalence estimates compared to bead beating for 5 seconds. The infection prevalence by 10 second bead beating was not statistically significantly different from the microscopy estimate, differences between these conditions probably reflecting variation in infection prevalences in batches of mosquitoes.

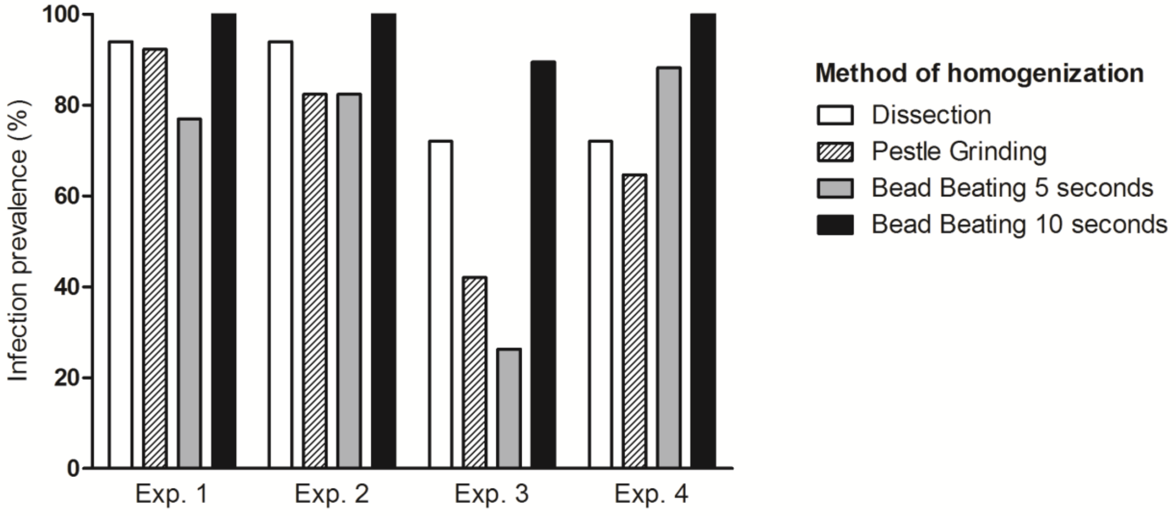

Supporting Figure S2. Processing of mosquitoes by CS-ELISA when tested for both *P. falciparum* and *P. vivax*

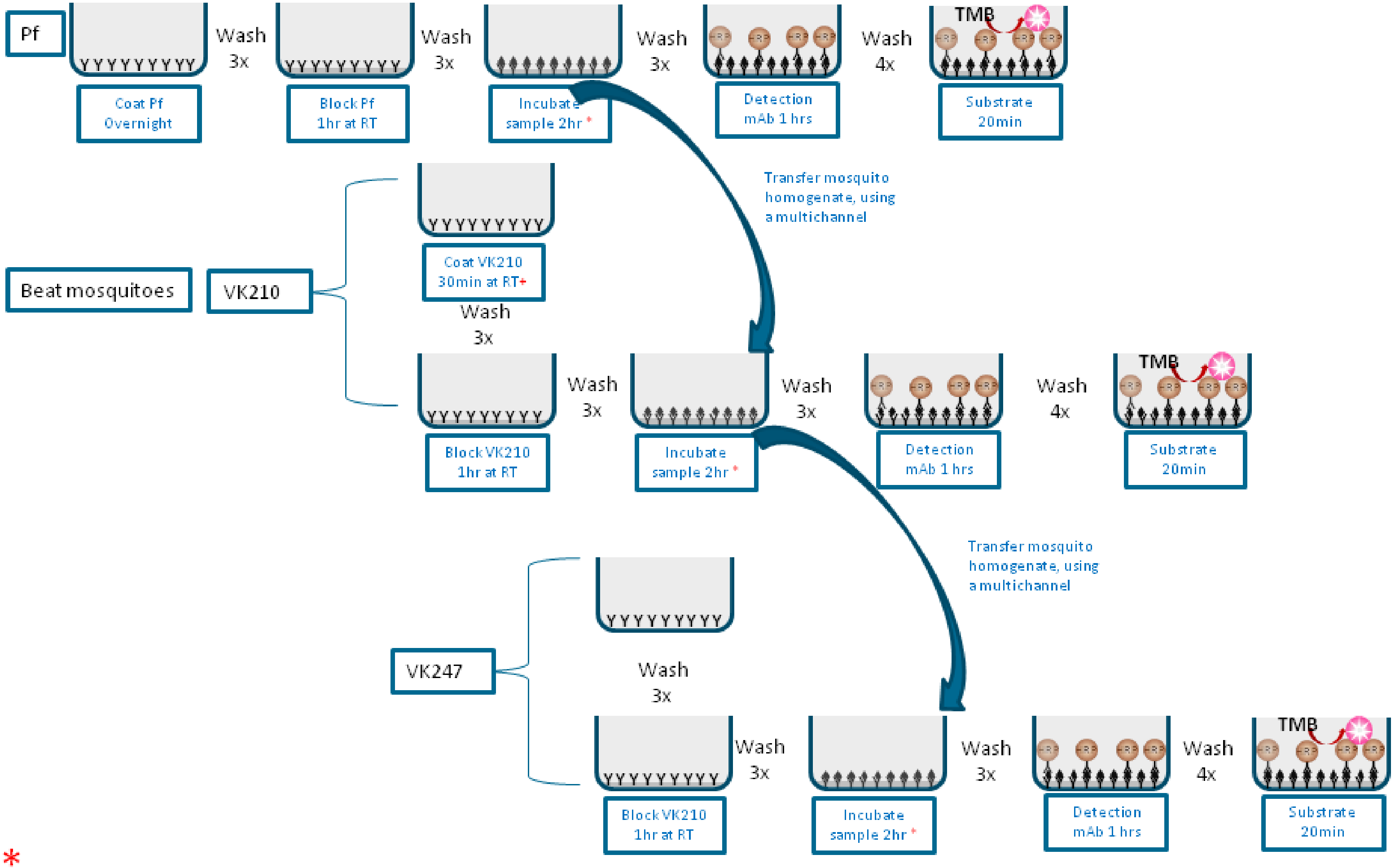

* Make sure that the 'labeled antibody solution' was prepared before the ' sample incubation period' of the Pf ends and the same for the VK210 plate is finished.

\+ do this right before incubating the previous sample
